# The Effect of Retained Hardware on Failure Among Prosthetic Joint Infections of the Knee in the Presence and Absence of *Staphylococcus aureus*

**DOI:** 10.1093/ofid/ofae306

**Published:** 2024-05-31

**Authors:** Justin J Kim, HeeEun Kang, Kathleen O Stewart

**Affiliations:** Section of Infectious Disease and International Health, Dartmouth Hitchcock Medical Center, Lebanon, New Hampshire, USA; Geisel School of Medicine at Dartmouth College, Hanover, New Hampshire, USA; Collaborative Healthcare-associated Infection Prevention Program, Dartmouth Hitchcock Medical Center, Lebanon, New Hampshire, USA; Section of Infectious Disease and International Health, Dartmouth Hitchcock Medical Center, Lebanon, New Hampshire, USA; Geisel School of Medicine at Dartmouth College, Hanover, New Hampshire, USA; The Dartmouth Institute for Health Policy and Clinical Practice, Dartmouth Hitchcock Medical Center, Lebanon, New Hampshire, USA; Collaborative Healthcare-associated Infection Prevention Program, Dartmouth Hitchcock Medical Center, Lebanon, New Hampshire, USA; Quality Assurance and Safety, Dartmouth Hitchcock Medical Center, Lebanon, New Hampshire, USA; Quality Assurance and Safety, Dartmouth Health, Lebanon, New Hampshire, USA

**Keywords:** debridement, antibiotics, and implant retention, 1-stage revision, 2-stage revision, effect modification

## Abstract

**Background:**

The risk of failure associated with different surgical strategies for prosthetic joint infections (PJIs) among patients with and without *Staphylococcus aureus* is uncertain. The purpose of this study was to assess whether *S. aureus* modifies the association between retained hardware and failure following revision surgery for PJI of the knee.

**Methods:**

This was a single-center retrospective cohort study of 106 first PJIs of the knee between 2016 and 2020 at a rural academic medical center. The exposure was retained hardware following revision surgery for PJI, and the outcomes were recurrent infection, any infection, and a composite outcome including any infection, unplanned revision, failure to undergo reimplantation, amputation, or death within 2 years of revision. We used negative binomial regression to quantify the association between the exposure and outcome and to assess the presence of *S. aureus* as an effect modifier.

**Results:**

Retained hardware was significantly associated with failure when defined as recurrent infection among *S. aureus* PJI (adjusted risk difference [aRD], 0.38; 95% CI, 0.12–0.64) but not in the absence of *S. aureus* (aRD, −0.02; 95% CI, −0.17 to 0.13), and *S. aureus* was an effect modifier (*P*_interaction_ = .01).

**Conclusions:**

We report a significant association between the presence of retained hardware and recurrent infection among *S. aureus* PJI of the knee, but not for non–*S. aureus* PJI. This could help inform the surgical management of PJI of the knee in cases where the microbiology is known before surgery.

Prosthetic joint infections (PJIs) are a devastating complication of joint replacement surgery. The treatment of PJIs is complicated, typically involving a combination of surgery (eg, debridement, antibiotics, and implant retention [DAIR] or 2-stage revision, and less commonly 1-stage revision with the exchange of some or all components of the prosthesis) and antibiotics (eg, oral vs intravenous, longer vs shorter duration) [1, 2]. While having retained hardware (ie, with a DAIR or 1-stage revision) is generally felt to have a stronger association with failure than hardware removal (ie, with a 2-stage revision), there are very few controlled studies (ie, with cohorts of patients with and without retained hardware) that rigorously describe the association between surgical strategies and failure. Some studies show that PJIs with retained hardware are more likely to fail than PJIs with hardware removal [3, 4], though others show no difference [5, 6]. This discrepancy may be explained in part by the varying definitions of failure commonly used in the PJI literature, some using microbiologic definitions of failure [3, 7, 8] and others defining failure as a composite outcome [9, 10]. Additionally, an increasing number of studies has shown that PJIs with *Staphylococcus aureus* are more likely to fail than PJIs with other organisms, whether or not hardware is removed [11–13]. However, the current literature also suggests that PJIs with *S. aureus* are more likely to fail in the presence of retained hardware [1, 2, 14, 15], likely owing to the persistence of biofilms [16].

The interplay between the presence of retained hardware, failure, and *S. aureus* is complex but clinically relevant in the management of PJIs, though few if any studies have examined the relationship between these variables precisely. For example, no study to our knowledge addresses the following question: Among *S. aureus* PJIs of the knee, how much more likely is DAIR to fail compared with a 2-stage revision? Thus, the purpose of this exploratory study was to assess the association between the presence of retained hardware and failure following revision surgery for PJI of the knee in the presence or absence of *S. aureus* and to determine how these associations differ across varying definitions of failure. We hypothesized that the association between retained hardware and failure would be more pronounced in the presence of *S. aureus*.

## METHODS

### Study Setting and Design

We identified 387 unique patients aged >18 years undergoing revision knee arthroplasty procedures at a 422-bed rural academic medical center between January 2016 and December 2020 using current procedural terminology codes and the *International Classification of Disease,* 10th Revision, procedure coding system as specified by the National Healthcare Safety Network [17]. We identified PJIs by manually reviewing the medical record of each patient and excluded 231 patients with no infectious disease notes and an additional 35 patients for whom the infectious disease notes did not mention any knee infection. Another 15 patients with a prior PJI in the same knee were excluded. The remaining 106 patients comprised our cohort of first PJIs of the knee. This study was determined to be not human research by the Institutional Review Board of the Dartmouth Hitchcock Office of Research Operations.

### Exposure, Outcome, and Covariates

The primary exposure was the presence of retained hardware following revision surgery for PJI, which we ascertained by manual review of operative notes. We used 3 definitions of failure as our primary outcomes including a diagnosis of recurrent infection in the same knee (ie, matching organism with identical antibiotic susceptibilities), a diagnosis of any infection in the same knee (ie, different organism, or matching organism with different antibiotic susceptibilities), and a composite outcome including a diagnosis of any infection, unplanned revision, failure to undergo reimplantation, amputation, or death from any cause. All outcomes were ascertained by manual review of the medical record within 2 years of revision surgery for the PJI. Patient-specific covariates at the time of revision surgery included age, sex, race, body mass index, diabetes, smoking history, indication for initial knee replacement, time to PJI, duration of symptoms, microbiology, operative findings (ie, cloudy, purulent, fistula, dehiscence, necrosis, synovitis), and antibiotic name, route, and planned duration. A complete data dictionary is provided in Supplementary Table 1.

### Statistical Analysis

We examined the frequency of categorical variables, distribution of continuous variables, and missingness of all variables by univariate analysis. By bivariate analysis, we determined the crude associations between the exposure and outcomes, and covariates and exposure. We also determined the crude associations between planned antibiotic duration, the use of a highly bioavailable or biofilm-active antibiotic (ie, fluoroquinolones, tetracyclines, trimethoprim-sulfamethoxazole, linezolid, clindamycin, metronidazole, or rifampin for at least 2 weeks), and outcomes among patients with retained hardware. We compared the medians of continuous variables using the Wilcoxon rank-sum test and the proportions of categorical variables using the Pearson χ^2^ or Fisher exact test, as appropriate.

We used negative binomial regression to determine the risk difference of failure in the presence of retained hardware compared with no retained hardware (ie, the absolute risk reduction, or proportion of failures among those with retained hardware minus the proportion of failures among those without retained hardware). The statistical model of the association between retained hardware, infection with *S. aureus*, and failure is given in Equation 1.


(1) $$\begin{array}{rl} \mathrm{risk}(\mathrm{failure})\;= & \;\beta_{0}\;+\;\beta_{1}\times (\text{retained hardware})\\ & +\;\beta_{2}\times (S.aureus)\\ & +\;\beta_{3}\times (\text{retained hardware})\times (S.aureus) \end{array}$$

Effect modification is a well-described phenomenon in epidemiology where the association between an exposure and outcome varies significantly in the presence of a third variable because of a biologically plausible mechanism (eg, the biofilms of *S. aureus* are more difficult to eradicate in the presence of hardware). We assessed whether the presence of *S. aureus* modified the association between the exposure and outcomes on the risk difference scale. Specifically, we used the statistical significance of the regression coefficient of the interaction term (ie, β_3_) to determine if the risk difference in the presence of *S. aureus* differed significantly from the risk difference in the absence of *S. aureus*. A full derivation of the risk differences in the presence and absence of *S. aureus* using Equation 1 is included in Supplementary Text 1. The following covariates were selected a priori as being the most plausible confounders (ie, most likely to influence the surgical plan and the presence of retained hardware, while also being associated with failure, but not in the causal pathway between the exposure and outcome): age >65 years, body mass index >35, diabetes, current smoking, and acute (ie, time to PJI <30 days or duration of symptoms <30 days, without arthrocutaneous fistula) vs chronic PJI (ie, time to PJI >30 days and duration of symptoms >30 days, or with arthrocutaneous fistula). We adjusted for confounding by these covariates using inverse probability of treatment weighting (IPTW) [18]. We assessed the covariate balance before and after applying the IPTWs using standardized mean differences. We also conducted secondary analyses assuming the “worst case” for losses to follow-up (ie, assuming failure for losses to follow up with no retained hardware), restricting the analysis to PJIs with positive cultures, combining *S. aureus* and *S. lugudnensis* infections, and combining all staphylococcal infections (ie, *S. aureus*, *S. lugdunensis*, and coagulase-negative staphylococci). We used SASStudio 3.8 software (SAS Institute, Cary, NC, USA) for all statistical analyses. We used an α of .05 as the significance threshold for all statistical tests.

## RESULTS

Demographic and clinical characteristics by the presence of retained hardware are given in Table 1. Of the 58 patients with retained hardware, 53 underwent DAIR and 5 underwent single-stage revision, while 48 patients underwent 2-stage revision and had no retained hardware. There were 9 (8%) losses to follow-up within 2 years of revision surgery. All patients in this cohort were White. Those with retained hardware were older (71 years old vs 65 years old; *P* < .01), experienced a PJI sooner after their initial knee replacement (1.5 years vs 4.1 years; *P* = .01), had a shorter duration of symptoms before the PJI (3 days vs 31 days; *P* < .01), more frequently had acute PJI (84% vs 44%; *P* < .01), had operative findings of dehiscence (12% vs 0%; *P* = .02), had longer planned antibiotic duration (40 weeks vs 6 weeks; *P* < .01), and were more likely to be prescribed antibiotics indefinitely (45% vs 0%; *P* < .01) compared with those with no retained hardware. Among patients with retained hardware, an antibiotic duration >12 weeks, indefinite antibiotics, and use of a highly bioavailable or biofilm-active antibiotic for >2 weeks were not significantly associated with any study outcomes (Supplementary Table 2).

**Table 1.** Demographic and Clinical Characteristics of Patients With First PJI of the Knee, 2016–2020

Covariate	Total, No. (%), n = 106	Retained Hardware, No. (%), n = 58^a^	No Retained Hardware, No. (%), n = 48	*P* Value^b^
Age, y^c^	68 (15)	71 (14)	65 (15)	<.01
Age >65 y	66 (62)	41 (71)	25 (52)	.05
Male	52 (49)	29 (50)	25 (52)	.83
White	106 (100)	58 (100)	48 (100)	–
BMI, kg/m^2^	34 (12)	33 (9)	35 (13)	.37
BMI >35 kg/m^2^	46 (43)	21 (36)	25 (52)	.10
Diabetes	32 (30)	16 (28)	16 (33)	.52
Current Smoker	8 (8)	2 (3)	6 (13)	.14
Reason for Initial Knee Replacement				
Osteoarthritis	77 (73)	38 (66)	39 (81)	.07
Trauma	4 (4)	3 (5)	1 (2)	.62
Cancer	1 (1)	1 (2)	0 (0)	1.00
Hardware Failure	20 (19)	13 (22)	7 (15)	.31
Other^d^	4 (4)	3 (5)	1 (2)	.62
Time to PJI, y^c^	2.7 (6.5)	1.5 (5.4)	4.1 (6.3)	.01
Duration of Symptoms, d^c^	7 (29)	3 (6)	31 (105)	<.01
Acute PJI^e^	70 (66)	49 (84)	21 (44)	<.01
PJI Microbiology				
*Staphylococcus aureus*	25 (24)	13 (22)	12 (25)	.75
*Streptococcus* spp.	20 (19)	14 (34)	6 (13)	.13
CoNS	19 (18)	10 (17)	9 (19)	.84
*S. lugdunensis*	3 (3)	3 (5)	0 (0)	.25
Gram-negative	6 (6)	3 (5)	3 (6)	1.00
Anaerobe	1 (1)	0 (0)	1 (2)	.45
Polymicrobial^f^	9 (8)	6 (10)	3 (6)	.51
Negative	23 (22)	9 (16)	14 (29)	.09
Operative Findings				
Cloudy	38 (36)	17 (29)	21 (44)	.12
Purulent	46 (43)	26 (45)	20 (42)	.74
Fistula	10 (9)	5 (9)	5 (10)	.75
Dehiscence	7 (7)	7 (12)	0 (0)	.02
Necrosis	12 (11)	8 (14)	4 (8)	.38
Synovitis	18 (17)	9 (16)	9 (19)	.66
Planned Antibiotic Duration, wk^c^	6.6 (50)	40 (94)	6 (0.2)	<.01
Indefinite Antibiotics Planned	26 (25)	26 (45)	0 (0)	<.01

Abbreviations: BMI, body mass index; CoNS, coagulase-negative staphylococci; PJI, prosthetic joint infection.

^a^Debridement, antibiotics, and implant retention (n = 53), 1-stage revision (n = 5).

^b^Pearson χ^2^ or Fisher exact for categorical variables; Wilcoxon rank-sum test for continuous variables.

^c^Median (interquartile range) for continuous variables.

^d^Rheumatoid arthritis (n = 2), psoriatic arthritis (n = 1), pigmented villonodular synovitis (n = 1).

^e^Time to PJI <30 d or duration of symptoms <30 d, without arthrocutaneous fistula.

^f^ *S. aureus* in 4 patients with polymicrobial PJI, 2 with retained hardware, 2 without retained hardware.

Defining failure as recurrent infection, and among PJIs with *S. aureus*, the risk of failure among patients with retained hardware was 0.38 greater than that of patients without retained hardware (95% CI, 0.12–0.64), adjusting for age, body mass index, diabetes, current smoking, and late PJI. Retained hardware was not significantly associated with recurrent infection in the absence of *S. aureus* (adjusted risk difference, −0.02; 95% CI, −0.17 to 0.13). The presence of *S. aureus* significantly modified the association between the presence of retained hardware and failure (*P*_interaction_ = 0.01). When defining failure as any infection or as a composite outcome, the association between retained hardware and failure was not significant even after assessing for effect modification by the presence of *S. aureus* (Table 2). The results were similar in all secondary analyses (Supplementary Tables 4–7). IPTW improved the balance of potential confounding covariates between subjects with retained hardware vs not, as the standardized mean differences were all negligible, with values between −0.10 and 0.10 (Supplementary Table 3) [19].

**Table 2.** Association Between Retained Hardware and Failure^a^ Within 2 Years of Revision Surgery for PJI of the Knee, Stratified on Infection With *Staphylococcus aureus* at the Time of Revision

A, Failure is Recurrent Infection				
*S. aureus* Present	Failures With Retained Hardware, n/N (%)	Failures With No Retained Hardware, n/N (%)	aRD^b^ (95% CI)	*P*_interaction_
Yes	8/15^c^ (53)	1/14^d^ (7)	0.38 (0.12–0.64)	.01
No	4/43 (9)	5/34 (15)	−0.02 (−0.17 to 0.13)	

B, Failure is Any Infection				
*S. aureus* Present	Failures With Retained Hardware, n/N (%)	Failures With No Retained Hardware, n/N (%)	aRD (95% CI)	*P*_interaction_
Yes	9/15 (60)	5/14 (36)	0.22 (−0.09 to 0.53)	.22
No	9/43 (21)	8/34 (24)	−0.01 (−0.20 to 0.18)	

C, Failure is Any Infection, Unplanned Revision, Delayed Reimplantation, Amputation, or Death				
*S. aureus* Present	Failures With Retained Hardware, n/N (%)	Failures With No Retained Hardware, n/N (%)	aRD (95% CI)	*P*_interaction_
Yes	11/15 (73)	7/14 (50)	0.18 (−0.14 to 0.50)	.30
No	15/43 (35)	12/34 (35)	−0.03 (−0.27 to 0.21)	

Abbreviations: aRD, adjusted risk difference; PJI, prosthetic joint infection.

^a^Assuming no failure for losses to follow-up (n = 9).

^b^Adjusted for age >65 y, body mass index >35 kg/m^2^, diabetes, current smoking, and acute PJI (ie, time to PJI <30 d or duration of symptoms <30 d, without arthrocutaneous fistula).

^c^Thirteen monomicrobial, 2 polymicrobial.

^d^Twelve monomicrobial, 2 polymicrobial.

## DISCUSSION

We have shown a significant association between the presence of retained hardware and recurrent infection among patients with *S. aureus* PJIs of the knee, though this association was not significant in the absence of *S. aureus*. Thus, the presence of *S. aureus* modified the association between retained hardware and recurrent infection. Moreover, we found no significant association between the presence of retained hardware and any infection, or the composite outcome of any infection, unplanned revision, delayed reimplantation, amputation, or death, in the presence or absence of *S. aureus*. Thus, the presence of *S. aureus* did not modify the association between retained hardware and any infection or the composite outcome. While the microbiology of a PJI is not always known before the surgical plan is determined, our findings suggest that patients known to have *S. aureus* PJI of the knee could benefit from hardware removal, despite the morbidity associated with a 2-stage exchange. Our findings also support that patients with *S. aureus* PJI of the knee with retained hardware should be counseled about the ongoing risk of recurrent infection, and closer monitoring of these patients could be considered (eg, with 6–12-month follow-up rather than being discharged from the clinic). The negative association between retained hardware and the other definitions of failure may be attributable to the fact that having a second surgery in a 2-stage revision creates an additional opportunity for patients to have new infections, poor healing, and failure of hardware. Thus, physicians should more carefully consider the risks vs benefits of 2-stage revisions, especially for non–*S. aureus* PJIs. Rather, a more realistic outlook for patients may be that there is a 30%–40% risk of developing another infection, and less commonly unplanned revision, amputation, or death, irrespective of the surgical strategy.

It was difficult to find comparable studies in the literature examining the association between retained hardware and failure in the PJI literature, as very few studies have directly compared the outcomes of PJI following retained hardware vs hardware removal. Studies defining failure as recurrent infection [8] or any infection [3, 4] generally reported worse outcomes with retained hardware compared with hardware removal, and the proportions of failure in patients with hardware removal were lower than reported in our study. However, studies using a composite definition of failure [5, 6, 20] reported similar outcomes with retained hardware compared with hardware removal, and the proportions were similar to those reported in our study. Possible explanations for these discrepancies include inconsistencies in accounting for heterogeneity in these cohorts by key variables such as symptom chronicity and microbiology including *S. aureus*. We did find 2 single-arm studies suggesting that failures were more common in *S. aureus* PJI managed with DAIR compared with 2-stage revisions [14, 15]. Another prospective cohort reported that *S. aureus* was significantly associated with failure in PJIs managed with DAIR, but not with 2-stage exchange [5]. However, we found no studies explicitly examining *S. aureus* as an effect modifier of the association between retained hardware and failure, as done in the present study.

The primary strength of this study is that we conducted a detailed chart review to ascertain with precision the exposure, outcomes, microbiology, and many of the other covariates, from which we obtained complete data sets for 92% of subjects. Weaknesses of this study included its single-center design, yielding a small, relatively homogenous study population, which may not be generalizable to all settings. While the power of our study to support our negative findings is uncertain, it is notable that multiple secondary analyses yielded identical positive and negative results. Finer but relevant details such as surgical technique (eg, depth and extent of debridement) could not easily be ascertained from chart review. In this retrospective study, we attempted to adjust for relevant confounders that could influence both the surgical strategy following PJI and failure, though residual confounding (eg, patient and surgeon preference) may persist. Notably, covariate balance after IPTW was improved without significant residual imbalances. We also restricted our study population to first PJIs so that the groups would be more comparable. Our data were not powered to conduct mediation analyses for antibiotic choice and duration, though the subgroup analysis of patients with retained hardware uncovered no association between failure and antibiotic duration >12 weeks, indefinite antibiotics, or using a highly bioavailable or biofilm-active agent for >2 weeks, mirroring the findings of more recent literature [21].

Prognostication is of paramount importance to patients who have developed a PJI following joint replacement. Our exploratory study suggests that the surgical strategy, in combination with microbiology, can enhance a patient's understanding of their recovery on the backdrop of a clear and transparent discussion of how failure is defined. Many would cite the need for a randomized controlled trial to understand the interplay of these variables, but the barriers to such trials are considerable [22]. While awaiting these trials to be conducted, studies similar to this could potentially be used to bolster our current understanding of what drives failure following PJI management, and to what extent.

## Supplementary Material

ofae306_Supplementary_Data

## Supplementary Data

Supplementary materials are available at *Open Forum Infectious Diseases* online. Consisting of data provided by the authors to benefit the reader, the posted materials are not copyedited and are the sole responsibility of the authors, so questions or comments should be addressed to the corresponding author.
