# Supplementary material for: The Effect of Retained Hardware on Failure Among Prosthetic Joint Infections of the Knee in the Presence and Absence of Staphylococcus aureus
Source: Open Forum Infect Dis. 2024 May 31;11(6):ofae306. doi: 10.1093/ofid/ofae306 (PMC11196895; doi:10.1093/ofid/ofae306)
Supplement: ofae306_Supplementary_Data [file ofae306_supplementary_data.docx]

**Supplemental Material**

| **Supplemental Table 1.** Data dictionary | | | |
| --- | --- | --- | --- |
| No. | Name | Definition | Source |
| 1 | Date of birth | – | NHSN |
| 2 | Age | [10] – [1] | Calculated |
| 3 | Sex | – | NHSN |
| 4 | Race | – | Patient banner |
| 5 | Body mass index | Weight/height^2^ (kg/m^2^) at time of [11] | NHSN |
| 6 | Diabetes | Diabetes diagnoses at time of [11] | NHSN |
| 7 | Current smoker | Current smoking noted at time of [11] | Any note |
| 8 | Reason for initial knee  replacement | – | Orthopaedic clinic  notes, operative notes |
| 9 | Date of initial knee  replacement | – | Orthopaedic clinic  notes, operative notes |
| 10 | Date of first PJI | Date of first synovial fluid, synovial, deep tissue, or bone culture coinciding with ID evaluation for suspected PJI | ID notes, microbiology |
| 11 | Date of revision surgery  for first PJI | – | Operative notes |
| 12 | Time to PJI | [10] – [9] | Calculated |
| 13 | Duration of symptoms | – | Orthopaedic clinic  notes, operative  notes, ID notes |
| 14 | PJI microbiology | Bacteria recovered from synovial fluid, synovium, deep tissue, or bone | Microbiology |
| 15 | Operative findings | – | Operative notes |
| 16 | Antibiotic end date | – | ID notes |
| 17 | Planned antibiotic  duration | [15] – [11], 2 years if [17]=Yes or Likely | Calculated |
| 18 | Indefinite antibiotics  planned | Yes or Likely^1^ | ID notes |
| 19 | Highly bioavailable or  biofilm active  antibiotics | Fluoroquinolones, tetracyclines, trimethoprim-sulfamethoxazole, linezolid, clindamycin, metronidazole, rifampin for at least 2 weeks | ID notes |
| 20 | Recurrent infection | Synovial fluid culture with the same organism and susceptibility as the first PJI within [11]+2 years | Microbiology |
| 21 | Any infection | Any positive synovial fluid culture within [11]+2 years | Microbiology |
| 22 | Unplanned revision | Any surgery not related to infection or reimplantation within [11]+2 years | Operative notes |
| 23 | Delayed reimplantation | Failure to have reimplantation following removal of hardware within [11]+2 years | Operative notes |
| 24 | Death | Date of death recorded within [11]+2 years | Patient banner, any note |
| 25 | Lost to follow up | Most recent result (eg, lab draw) or clinical encounter (eg, clinic visit) recorded within [11]+2 years | Results, any note |
| NHSN=National Healthcare Safety Network, PJI=prosthetic joint infection, ID=infectious diseases  ^1^Yes – Indefinite suppression stated in ID notes, prescription on record >2 years later; No – Antibiotic end date explicitly stated in ID notes; Likely – Indefinite suppression mentioned in ID notes, but not prescribed due to [19], [20], [21], [22], or [23] within [11]+2 years; Unlikely – Indefinite suppression not mentioned in ID notes, not prescribed due to [19], [20], [21], [22] or [23] within [11]+2 years | | | |

**Supplemental Text 1.** Derivation of risk differences in the presence and absence of *S. aureus*

For the model

risk(failure)=β_0_ + β_1_(retained hardware) + β_2_(*S. aureus*) + β_3_(retained hardware)(*S. aureus*)

risk difference(failure|*S. aureus*=1)

=risk(failure|retained hardware=1, *S. aureus=*1) – risk(failure|retained hardware=0, *S. aureus*=1)

={β_0_ + β_1_(1) + β_2_(1) + β_3_(1)(1)} – {β_0_ + β_1_(0) + β_2_(1) + β_3_(0)(1)}

=β_1_ + β_3_

where A|B is “A given B”

risk difference(failure|*S. aureus*=0)

=risk(failure|retained hardware=1, *S. aureus=*0) – risk(failure|retained hardware=0, *S. aureus*=0)

={β_0_ + β_1_(1) + β_2_(0) + β_3_(1)(0)} – {β_0_ + β_1_(0) + β_2_(0) + β_3_(0)(0)}

=β_1_

If the 95% confidence interval of (β_1_ + β_3_) does not cross zero, then there is a significant difference in the proportion of failures among patients with retained hardware compared to those with no retained hardware in the presence of *S. aureus*.

If the 95% confidence interval of β_1_ does not cross zero, then there is a significant difference in the proportion of failures among patients with retained hardware compared to those with no retained hardware in the absence of *S. aureus*.

If the 95% confidence interval of β_3_ does not cross zero (ie, p-interaction <0.05), then the presence of *S. aureus* modifies the effect of retained hardware on failure, on the risk difference scale.

| **Supplemental Table 2.** Outcomes among patients with retained hardware within 2 years of revision surgery for first PJI of the knee | | | | |
| --- | --- | --- | --- | --- |
| A. | Antibiotic duration >12 weeks | | |  |
|  | Yes,  No. (%), n=42 | | No,  No. (%), n=16 | p-value^1^ |
| Recurrent infection | 9 (21) | | 3 (19) | 1.00 |
| Any infection | 13 (31) | | 5 (31) | 1.00 |
| Any infection, unplanned revision,  delayed reimplantation, amputation,  or death | 17 (40) | | 9 (56) | 0.28 |
| B. | Antibiotic duration indefinite | | |  |
|  | Yes,  No. (%), n=26 | No,  No. (%), n=32 | | p-value |
| Recurrent infection | 5 (19) | 7 (22) | | 0.80 |
| Any infection | 8 (31) | 10 (31) | | 0.97 |
| Any infection, unplanned revision,  delayed reimplantation, amputation,  or death | 11 (42) | 15 (47) | | 0.73 |
| C. | Treatment with highly bioavailable or biofilm active antibiotic for >2 weeks | | |  |
|  | Yes,  No. (%), n=32 | No,  No. (%), n=26 | | p-value |
| Recurrent infection | 9 (28) | 3 (12) | | 0.12 |
| Any infection | 12 (38) | 6 (23) | | 0.24 |
| Any infection, unplanned revision,  delayed reimplantation, amputation,  or death | 15 (47) | 11 (42) | | 0.73 |
| PJI=prosthetic joint infection,  ^1^Pearson χ^2^ or Fisher exact | | | | |

| **Supplemental Table 3.** Covariate balance before and after IPTW | | |
| --- | --- | --- |
|  | Standardized mean difference | |
| Covariate | Before IPTW | After IPTW |
| Age >65 years | 0.39 | –0.08 |
| Body mass index >35 kg/m^2^ | –0.32 | –0.02 |
| Diabetes | –0.13 | 0.01 |
| Current smoking | –0.34 | 0.01 |
| Early PJI^1^ | 0.94 | –0.02 |
| IPTW=inverse probability of treatment weighting  ^1^time to PJI <30 days or duration of symptoms <30 days, without arthrocutaneous fistula | | |

| **Supplemental Table 4.** Association between retained hardware and failure within 2 years of revision surgery for PJI of the knee, stratified on infection with *Staphylococcus aureus* at the time of revision, assuming failure for losses to follow up with no retained hardware^1^ | | | | |
| --- | --- | --- | --- | --- |
| A. Failure is recurrent infection | | | | |
| *S. aureus*  present | Failures with  retained hardware, n/N (%) | Failures with  no retained hardware,  n/N (%) | aRD^2^ (95% CI) | p-interaction |
| Yes | 8/15 (53%) | 1/14 (7%) | 0.38 (0.12, 0.64) | <0.01 |
| No | 4/43 (9%) | 8/34 (24%) | -0.13 (-0.32, 0.05) |  |
| B. Failure is any infection | | | | |
| *S. aureus*  present | Failures with  retained hardware,  n/N (%) | Failures with  no retained hardware,  n/N (%) | aRD (95% CI) | p-interaction |
| Yes | 9/15 (60%) | 5/14 (36%) | 0.22 (-0.09, 0.53) | 0.07 |
| No | 9/43 (21%) | 11/34 (32%) | -0.12 (-0.33, 0.09) |  |
| C. Failure is any infection, unplanned revision, delayed reimplantation, amputation, or death | | | | |
| *S. aureus*  present | Failures with  retained hardware,  n/N (%) | Failures with  no retained hardware,  n/N (%) | aRD (95% CI) | p-interaction |
| Yes | 11/15 (73%) | 7/14 (50%) | -0.14 (-0.14, 0.50) | 0.14 |
| No | 15/43 (35%) | 14/34 (41%) | -0.12 (-0.36, 0.12) |  |
| PJI=prosthetic joint infection, aRD=adjusted risk difference, CI=confidence interval  ^1^n=3  ^2^adjusted for age >65 years, body mass index >35 kg/m^2^, diabetes, current smoking, and acute PJI (ie, time to PJI <30 days or duration of symptoms <30 days, without arthrocutaneous fistula) | | | | |

| **Supplemental Table 5.** Association between retained hardware and failure^1^ within 2 years of revision surgery for PJI of the knee, stratified on infection with *Staphylococcus aureus* at the time of revision, restricted to PJI with positive cultures | | | | |
| --- | --- | --- | --- | --- |
| A. Failure is recurrent infection | | | | |
| *S. aureus*  present | Failures with  retained hardware, n/N (%) | Failures with  no retained hardware,  n/N (%) | aRD^2^ (95% CI) | p-interaction |
| Yes | 8/15 (53%) | 1/14 (7%) | 0.43 (0.15, 0.70) | 0.02 |
| No | 3/34 (9%) | 1/20 (5%) | 0.06 (-0.08, 0.19) |  |
| B. Failure is any infection | | | | |
| *S. aureus*  present | Failures with  retained hardware,  n/N (%) | Failures with  no retained hardware,  n/N (%) | aRD (95% CI) | p-interaction |
| Yes | 9/15 (60%) | 5/14 (36%) | 0.28 (-0.04, 0.60) | 0.32 |
| No | 8/34 (24%) | 4/20 (20%) | 0.07 (-0.15, 0.30) |  |
| C. Failure is any infection, unplanned revision, delayed reimplantation, amputation, or death | | | | |
| *S. aureus*  present | Failures with  retained hardware,  n/N (%) | Failures with  no retained hardware,  n/N (%) | aRD (95% CI) | p-interaction |
| Yes | 11/15 (73%) | 7/14 (50%) | 0.24 (-0.08, 0.58) | 0.13 |
| No | 10/34 (29%) | 7/20 (35%) | -0.09 (-0.38, 0.20) |  |
| PJI=prosthetic joint infection, aRD=adjusted risk difference, CI=confidence interval  ^1^assuming no failure for losses to follow up (n=9)  ^2^adjusted for age >65 years, body mass index >35 kg/m^2^, diabetes, current smoking, and acute PJI (ie, time to PJI <30 days or duration of symptoms <30 days, without arthrocutaneous fistula) | | | | |

| **Supplemental Table 6.** Association between retained hardware and failure^1^ within 2 years of revision surgery for PJI of the knee, stratified on infection with *Staphylococcus aureus* or *S. lugdunensis* at the time of revision | | | | |
| --- | --- | --- | --- | --- |
| A. Failure is recurrent infection | | | | |
| *S. aureus* or *lugdunensis*  present | Failures with  retained hardware, n/N (%) | Failures with  no retained hardware,  n/N (%) | aRD^2^ (95% CI) | p-interaction |
| Yes | 8/18 (44%) | 1/15 (7%) | 0.32 (0.08, 0.57) | 0.02 |
| No | 4/40 (10%) | 5/33 (15%) | -0.02 (-0.18, 0.14) |  |
| B. Failure is any infection | | | | |
| *S. aureus* or *lugdunensis*  present | Failures with  retained hardware,  n/N (%) | Failures with  no retained hardware,  n/N (%) | aRD (95% CI) | p-interaction |
| Yes | 9/18 (50%) | 6/15 (40%) | 0.12 (-0.17, 0.42) | 0.63 |
| No | 9/40 (23%) | 7/33 (21%) | 0.03 (-0.15, 0.22) |  |
| C. Failure is any infection, unplanned revision, delayed reimplantation, amputation, or death | | | | |
| *S. aureus* or *lugdunensis*  present | Failures with  retained hardware,  n/N (%) | Failures with  no retained hardware,  n/N (%) | aRD (95% CI) | p-interaction |
| Yes | 11/18 (61%) | 8/15 (53%) | 0.07 (-0.23, 0.38) | 0.77 |
| No | 15/40 (38%) | 11/33 (33%) | 0.02 (-0.23, 0.26) |  |
| PJI=prosthetic joint infection, aRD=adjusted risk difference, CI=confidence interval  ^1^assuming no failure for losses to follow up (n=9)  ^2^adjusted for age >65 years, body mass index >35 kg/m^2^, diabetes, current smoking, and acute PJI (ie, time to PJI <30 days or duration of symptoms <30 days, without arthrocutaneous fistula) | | | | |

| **Supplemental Table 7.** Association between retained hardware and failure^1^ within 2 years of revision surgery for PJI of the knee, stratified on infection with *Staphylococcus aureus*, *S. lugdunensis,* or coagulase negative staphylococci at the time of revision | | | | |
| --- | --- | --- | --- | --- |
| A. Failure is recurrent infection | | | | |
| Staphylococci  present | Failures with  retained hardware, n/N (%) | Failures with  no retained hardware,  n/N (%) | aRD^2^ (95% CI) | p-interaction |
| Yes | 10/31 (32%) | 2/24 (8%) | 0.21 (0.03, 0.39) | 0.03 |
| No | 2/27 (7%) | 4/24 (17%) | -0.06 (-0.24, 0.11) |  |
| B. Failure is any infection | | | | |
| Staphylococci  present | Failures with  retained hardware,  n/N (%) | Failures with  no retained hardware,  n/N (%) | aRD (95% CI) | p-interaction |
| Yes | 12/31 (39%) | 9/24 (38%) | 0.02 (-0.21, 0.25) | 0.76 |
| No | 6/27 (22%) | 4/24 (17%) | 0.07 (-0.15, 0.29) |  |
| C. Failure is any infection, unplanned revision, delayed reimplantation, amputation, or death | | | | |
| Staphylococci  present | Failures with  retained hardware,  n/N (%) | Failures with  no retained hardware,  n/N (%) | aRD (95% CI) | p-interaction |
| Yes | 15/31 (48%) | 12/24 (50%) | 0.01 (-0.24, 0.26) | 0.86 |
| No | 11/27 (41%) | 7/24 (29%) | 0.05 (-0.25, 0.34) |  |
| PJI=prosthetic joint infection, aRD=adjusted risk difference, CI=confidence interval  ^1^assuming no failure for losses to follow up (n=9)  ^2^adjusted for age >65 years, body mass index >35 kg/m^2^, diabetes, current smoking, and acute PJI (ie, time to PJI <30 days or duration of symptoms <30 days, without arthrocutaneous fistula) | | | | |
